# Expression profiles of long noncoding RNAs and messenger RNAs in the border zone of myocardial infarction in rats

**DOI:** 10.1186/s11658-019-0185-6

**Published:** 2019-12-02

**Authors:** Qingkun Meng, Zhijun Sun, Hui Gu, Jiaying Luo, Jingjing Wang, Chuanhe Wang, Su Han

**Affiliations:** Shengjing Hospital, China Medical University, Shenyang, China

**Keywords:** Long noncoding RNAs, mRNAs, Myocardial infarction, Border zone, Area at risk, Co-expression network, Bioinformation

## Abstract

**Background:**

The participation of long noncoding RNAs (lncRNAs) in myocardial infarction has recently been noted. However, their underlying roles in the border zone of myocardial infarction remain unclear. This study uses microarrays to determine the profiles of lncRNAs and mRNAs in the border zone.

**Methods:**

Bioinformatics methods were employed to uncover their underlying roles. Highly dysregulated lncRNAs was further validated via PCR.

**Results:**

Four hundred seven lncRNAs and 752 mRNAs were upregulated, while 132 lncRNAs and 547 mRNAs were downregulated in the border zone of myocardial infarction. A circos graph was constructed to visualize the chromosomal distribution and classification of the dysregulated lncRNAs and mRNAs. The upregulated mRNAs in the border zone were most highly enriched in cytokine activity, binding, cytokine receptor binding and related processes, as ascertained through Go analysis. Pathway analysis of the upregulated mRNAs showed the most significant changes were in the TNF signaling pathway, cytokine–cytokine receptor interaction and chemokine signaling pathway and similar pathways and interactions. An lncRNA–mRNA co-expression network was established to probe into the underlying functions of the 10 most highly dysregulated lncRNAs based on their co-expressed mRNAs. In the co-expression network, we found 16 genes directly involved in myocardial infarction, including Alox5ap, Itgb2 and B4galt1. The lncRNAs AY212271, EF424788 and MRAK088538, among others, might be associated with myocardial infarction. BC166504 is probably a key lncRNA in the border zone of myocardial infarction.

**Conclusions:**

The results may have revealed some aberrantly expressed lncRNAs and mRNAs that contribute to the underlying pathophysiological mechanisms of myocardial infarction.

## Background

Myocardial infarction causes millions of deaths worldwide every year. The border zone of the myocardial infarction is of considerable interest. During myocardial infarction, certain changes in the border zone, including apoptosis, fibrosis and inflammation, play important roles in determining the chances of patient survival [1].

The impairment and recovery of cardiacmyocytes have both been linked to changes in gene expression [2]. LncRNAs are defined as noncoding RNA transcripts over 200 nt in length without protein-coding ability [3]. They are known to be involved in gene imprinting [4], cardiac development and differentiation [5, 6], cardiac hypertrophy [7, 8], myocardial infarction [9–13] and heart failure [14–18], among other processes of interest in cardiology. Their regulatory functions mainly depend on epigenetic regulation, transcriptional regulation, post-transcriptional gene regulation, competing endogenous RNAs, post-translational gene regulation on protein turnover and nuclear compartmentalization [19].

Genome-wide profiling of the cardiac transcriptome after myocardial infarction has been performed, revealing heart-specific long non-coding RNAs [9, 10]. Expression profiling and ontology analysis of lncRNAs in the post-ischemic heart have also been performed [12]. Wang et al. constructed a differential lncRNA–mRNA co-expression network in myocardial infarction [13]. Ishii et al. found a novel non-coding RNA, MIAT, the overexpression of which confers risk of myocardial infarction [11].

Although a few cardiology-focused lncRNA studies have been performed, the potential roles of lncRNAs in the border zone of myocardial infarction have received little attention. This study uses microarrays to determine the profiles of lncRNAs and mRNAs in the border zone.

## Methods

### Animals

The Wistar rats used in this experiment were obtained from Chang Sheng Biotechnology. This investigation was performed according to the protocols approved by the Medical Research and New Technology Ethical Committee of the Second Affiliated Hospital (Shengjing Hospital) of China Medical University (approval no. 2015PS295K).

### Myocardial infarction surgery

Adult male Wistar rats weighing 390.45 ± 51.45 g were anesthetized with an intraperitoneal injection of 10% chloralhydrate (3 mg/g). We created a myocardial infarction model by ligation of the left anterior descending artery (LAD) with a 6–0 silk suture. Sham-operated rats underwent an identical procedure without tying. Ligation was verified through observation of changes in the ECG and visualized as marked blanching of the left ventricle.

### Determination of the border zone of the infarct region

**Fig. 1:**
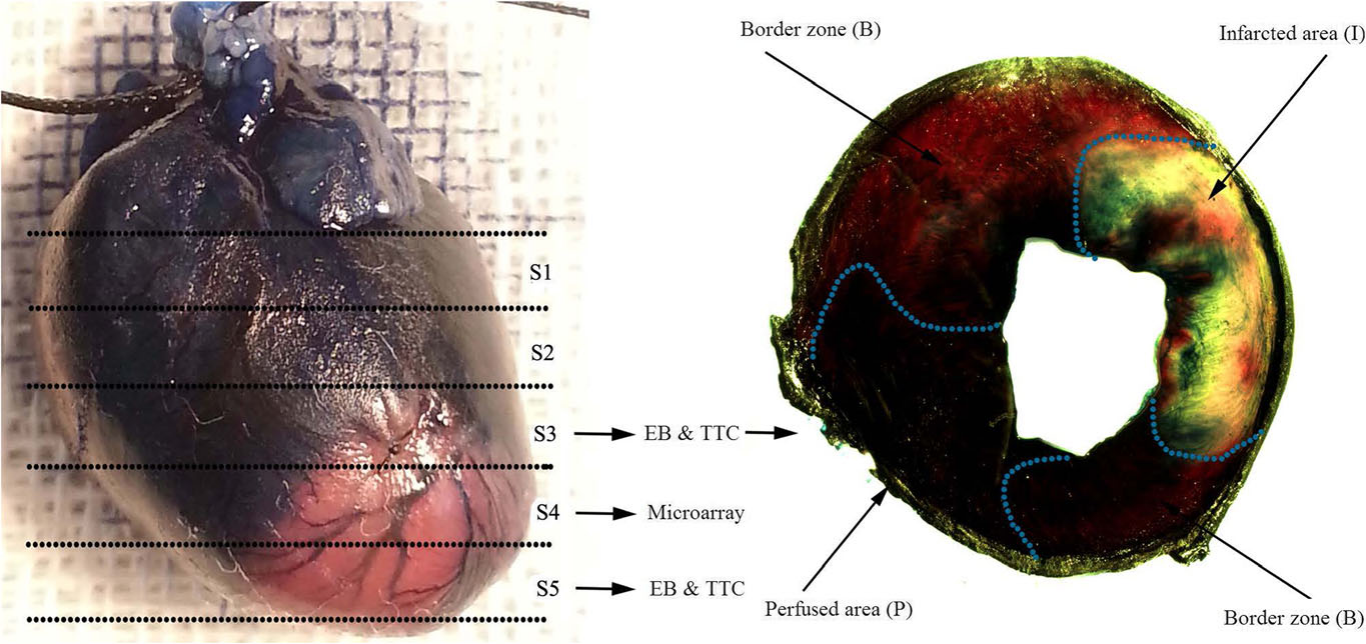
Slice 3 (S3) and slice 5 (S5) were double-stained with EB and TTC. The perfused area is deep blue, EB stained and TTC stained. The infarcted area is achromatous, EB unstained and TTC unstained. The border zone is red, EB unstained and TTC stained

The rats were killed 6 h after the procedure. Evans Blue dye (EB) and triphenyltetrazolium chloride (TTC) dual-dye staining was performed to precisely determine the border zone around the infarct region [20]. Five slices were cut equally from base to apex of the heart. The border zone and the infarct region were assessed by a blinded observer using computer-assisted planimetry. The border zone of the infarct region was identified as Evans blue unstained and TTC stained (red). Through comparison with the adjacent TTC section (slices 3 and 5), we localized the border zone of slice 4. Radial segments of slice 4 (the border zone) were used for microarray analysis and quantitative RT-PCR (Fig. 1).

### RNA extraction and quality control

LncRNA microarray analysis was performed by Kangchen Bio-tech using 3 samples from the infarction group and 3 samples from the sham operation group. RNA quantity and quality were measured with a NanoDrop ND-1000. The integrity of the RNA was assessed using standard denaturing agarose gel electrophoresis.

### Microarray analysis of lncRNA and mRNA expression

The Arraystar Rat LncRNA microarray (4 × 44 k) contains about 9000 lncRNAs from the databases of NCBI RefSeq and UCSC.

Sample labeling and array hybridization were performed according to the manufacturer’s protocol (Agilent Technology) with minor modifications. The hybridized arrays were washed, fixed and scanned. Agilent Feature Extraction software (version 11.0.1.1) was used to analyze the acquired array images. Quantile normalization and subsequent data processing were performed using the GeneSpring GX v12.1 software package (Agilent Technologies). After quantile normalization of the raw data, lncRNAs and mRNAs for which at least 3 out of 6 samples had flags in the categories Present or Marginal (All Targets Value) were chosen for further analysis.

All the microarray data have been submitted to GEO with the accession number GSE90745. They can also be accessed through the GEO platform with accession number GPL15690.

### Gene ontology and pathway analysis

Gene ontology (GO) and pathway analysis were applied to determine GO terms and/or the functions of these aberrantly expressed mRNAs in several biological pathways. GO analysis is used to determine processes or functional categories that are differentially expressed and mainly focuses on three aspects: biological processes (BP), molecular functions (MF) and cellular components (CC). To investigate the biological functions of differentially expressed mRNAs, we also searched the Kyoto Encyclopedia of Genes and Genomes (KEGG) pathway.

### Construction of the lncRNA-mRNA co-expression network

To identify the interaction network for lncRNAs and mRNAs, a co-expression network was constructed. The expression intensities of the lncRNAs and mRNAs were normalized. The relevance of each lncRNA–mRNA pair was calculated using Pearson’s correlation coefficient (PCC).

For a clear look at the most highly regulated lncRNAs and mRNAs, only the top 5 up- and downregulated lncRNAs and the top 10 up- and downregulated co-expressed mRNAs are presented in the visual network. Cytoscape 3.4.0 was used for visual representation of the network. In this network, nodes were lncRNAs or mRNAs, and when two nodes connected by an edge indicate they were co-expressed.

### Quantitative RT-PCR validation assay

Quantitative RT-PCR was performed to confirm the differentially expressed lncRNAs in the microarray analysis. Total RNA was extracted using Trizol agent (Invitrogen), then reverse-transcribed into cDNA by PrimeScript RT Reagent Kit with gDNA Eraser (TaKaRa) according to the manufacturers’ protocols. Real-time PCR was performed on an Applied Biosystems 7500 FAST Real-time PCR System using SYBR Premix Ex Taq II (TaKaRa). The specific primers were designed by Sangon Biotech. All experiments were performed in triplicate and normalized to β-actin. The median of each triplicate was used to calculate the relative levels of lncRNAs.

### Statistical methods

Data are expressed as the means ± standard deviation. Student’s t-test was performed for comparisons between two groups. Differences with *p* < 0.05 were considered statistically significant. The false discovery rate (FDR) was calculated to correct the *p*-value. Fold change > 2 and *p* < 0.05 were set as the threshold values to designate up- and downregulated lncRNAs and mRNAs.

## Results

### Expression profiles of lncRNAs and mRNAs in in the border zone of myocardial infarction

**Fig. 2:**
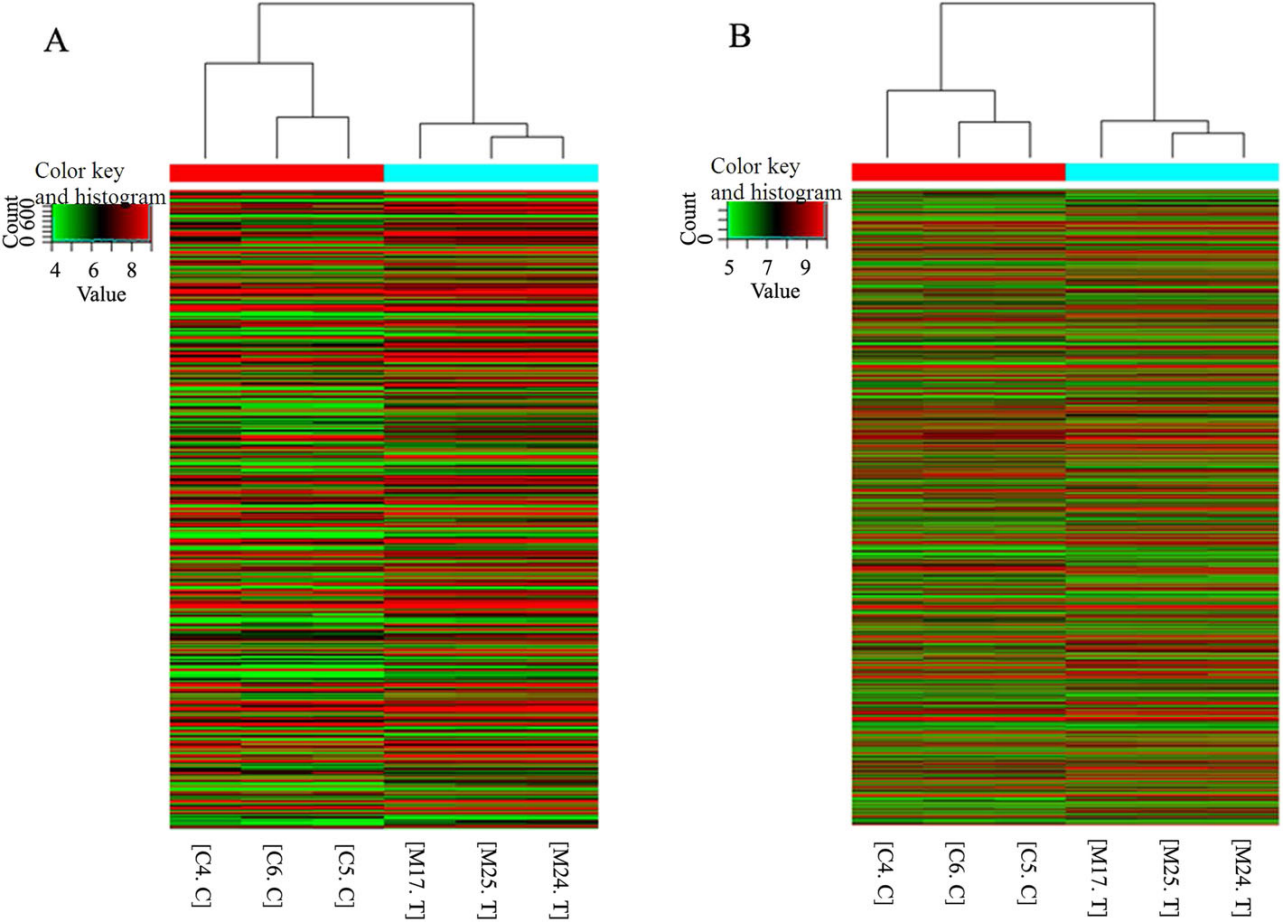
Heat map and hierarchical clustering of lncRNA (**a**) and mRNA (**b**) differential expression profiles between the border zone of myocardial infarction and the control zone of sham operation groups. “Red” indicates high relative expression, and “Green” indicates low relative expression

We performed a microarray analysis to obtain a global expression profile of lncRNAs and mRNAs in the border zone of myocardial infarction in Wistar rats. In total, 24,529 lncRNAs and mRNAs were detected in the array (Fig. 2). Of these, 407 lncRNAs were upregulated with a fold change > 2 and *p* < 0.05 compared with those in the sham operation group. MRuc008qhz, XR_006843, EF424788, BC166504 and AY212271 were the top five upregulated lncRNAs. In addition, 132 lncRNAs were downregulated with a fold change > 2 and *p* < 0.05. MRAK042828, BC089979, MRAK078284, AY539885 and MRAK088538 were the top five downregulated lncRNAs.

We found 752 upregulated mRNAs with a fold change > 2 and *p* < 0.05. The top five were NM_012589, NM_001109536, NM_053647, NM_001107589 and NM_019233. In addition, 547 mRNAs were downregulated with a fold change > 2 and *p* < 0.05. The top five were NM_012506, NM_031349, NM_022209, NM_001004131 and NM_001108163.

**Fig. 3:**
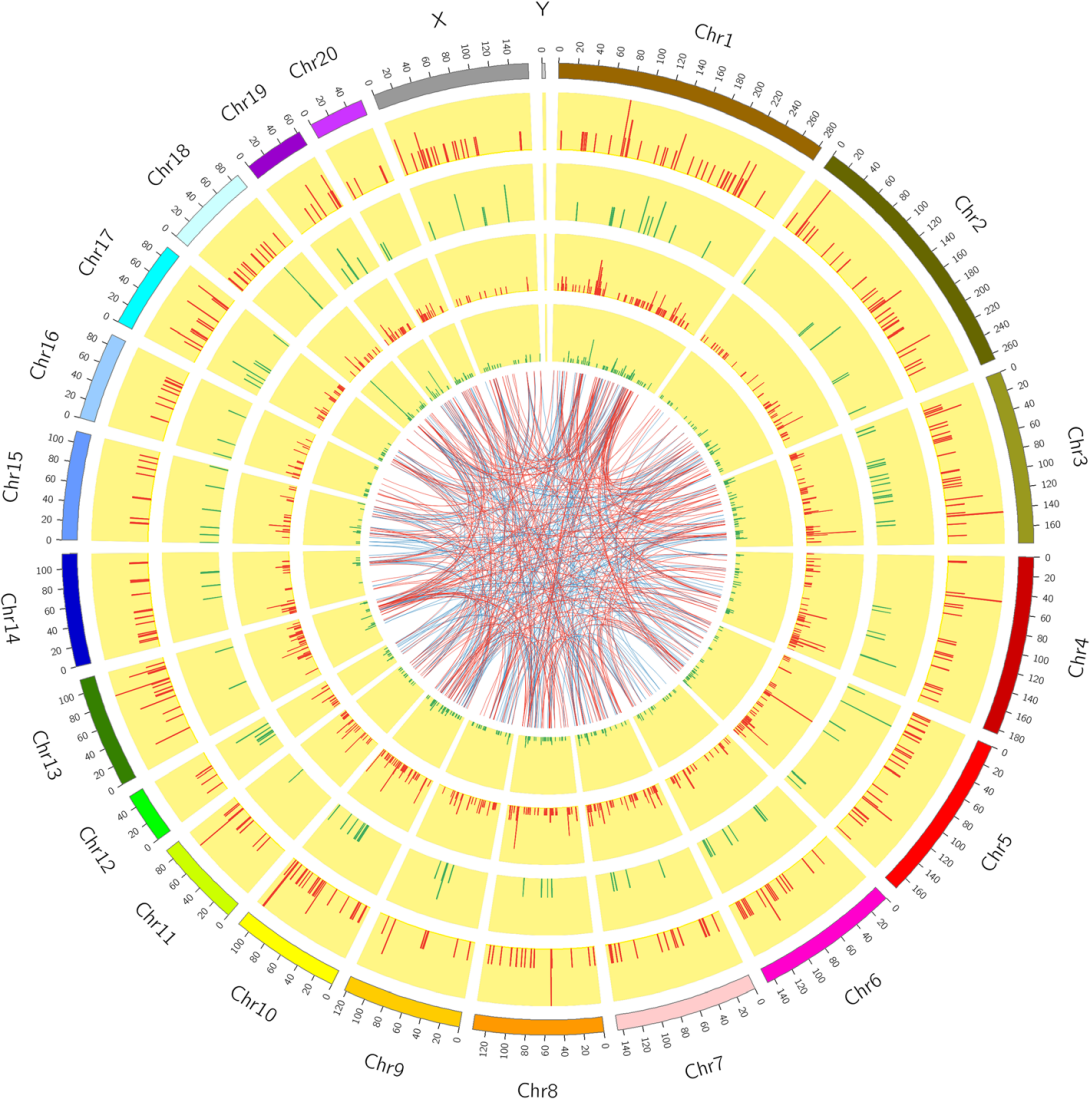
The outermost circle is the autosomal distribution map. The second and third circles are the distribution of differentially expressed genes on chromosomes. The red lines are upregulated and the green lines are downregulated. The higher the column, the more differentially expressed genes are in the region. The fourth and fifth circles are the distribution of differentially expressed lncRNAs on chromosomes. The expression form is related to the expression of RNA. The internal connection indicates that Top500 co-expresses the corresponding relationship between lncRNAs and mRNAs. Red indicates a positive correlation and blue indicates a negative correlation

We constructed a circos graph to visualize the chromosomal distribution and classification of the dysregulated lncRNAs and mRNAs (Fig. 3).

### GO and pathway analyses

To further investigate the functions of differentially expressed mRNAs identified from the border zone of myocardial infarction, we performed GO and pathway analyses. GO analysis provides a controlled vocabulary to describe differentially expressed transcript attributes in all organisms. Fisher’s exact test is used to find if there is more overlap between the differentially expressed list and the GO annotation list than would be expected by chance, and p denotes the significance of GO term enrichment in the differentially expressed genes. The lower the value of *p*, the more significant the GO term (*p* < 0.05 is recommended).

**Fig. 4:**
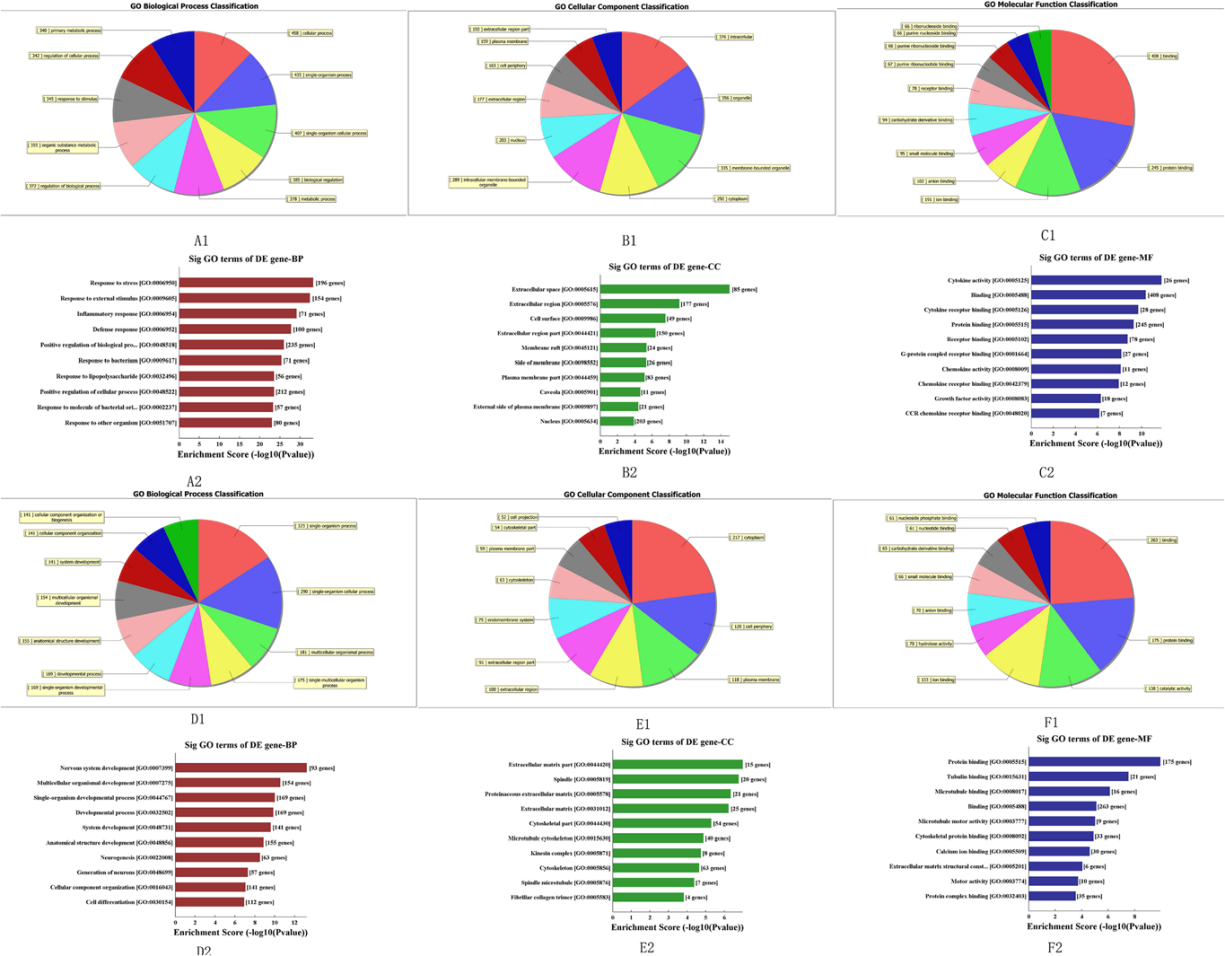
Pie charts indicate the top 10 gene quantity of GO terms. Bar charts indicate the top 10 enrichment scores of GO terms. **a**–**c** indicate biological process (BP), cellular component (CC) and molecular function (MF) of the upregulated mRNAs, **d**–**f** indicate the BP, CC and MF of the downregulated mRNAs. *p* < 0.05

In the border zone of myocardial infarction, the upregulated mRNAs were involved in 1638 biological processes (BP), 63 cellular components (CC) and 103 molecular functions (MF). The downregulated mRNAs were involved in 487 BP, 83 CC and 118 MF. In the BP category, the highest enrichment scores of the GO term for upregulated mRNAs were response to stress, while the highest for downregulated mRNAs were nervous system development. In the CC category, the most significant terms for upregulated mRNAs appeared in extracellular space, and for downregulated mRNAs appeared in extracellular matrix part. In the MF category, the most represented term for upregulated mRNAs was cytokine activity, and for downregulated mRNAs was protein binding (Fig. 4).

Pathway analysis was performed as a functional analysis mapping aberrantly expressed genes to KEGG pathways. The Fisher *p* value denotes the significance of the pathway correlated to the conditions. The lower the value, the. More significant the pathway (the recommended cutoff is 0.05).

**Fig. 5:**
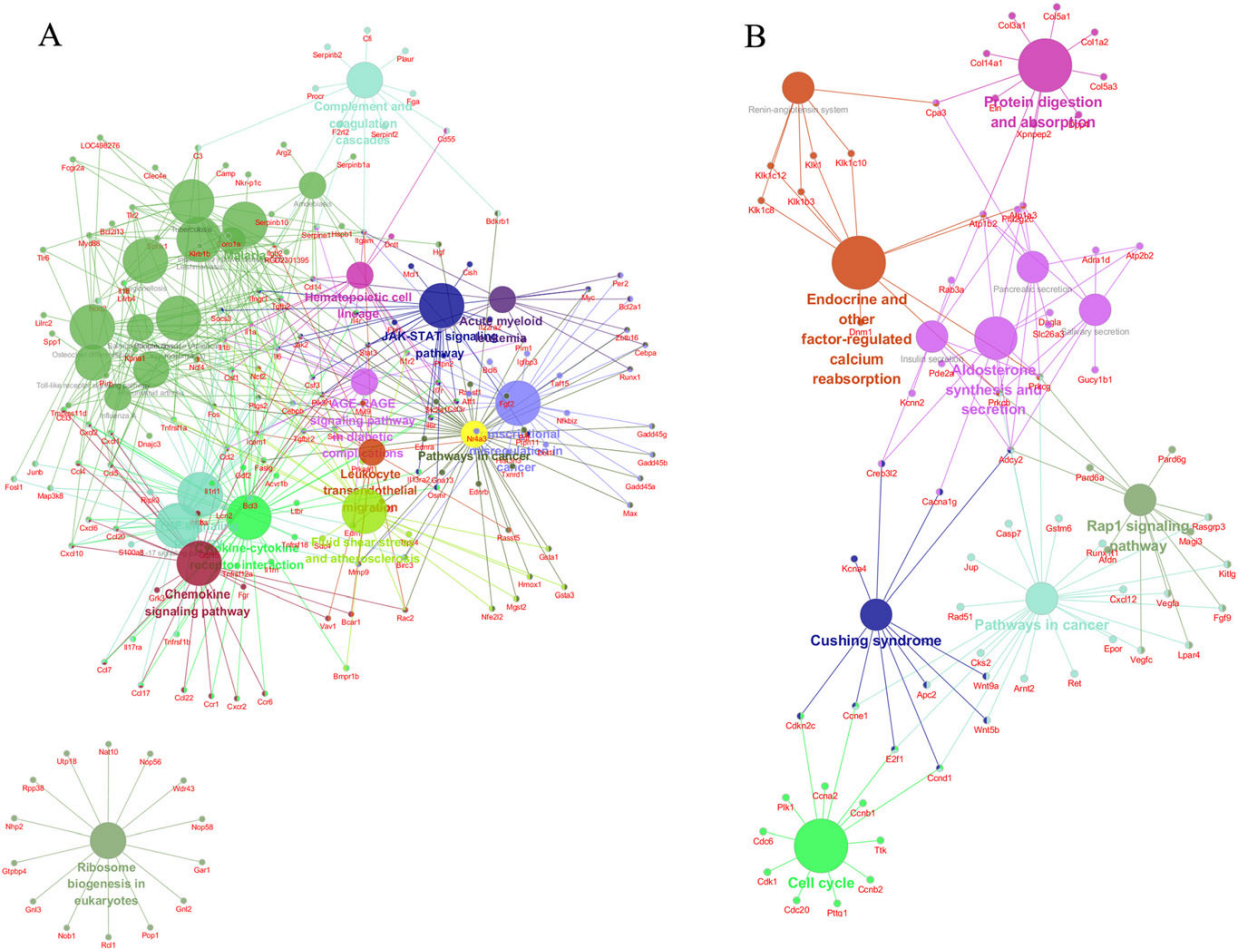
Kyoto Encyclopedia of Genes and Genomes (KEGG) pathway analysis of aberrantly expressed genes performed using ClueGO. **a** KEGG pathway classification of upregulated genes. **b** KEGG pathway classification of downregulated genes

In the border zone of myocardial infarction, the upregulated mRNAs were involved in 51 pathways and the downregulated genes were involved in 29 pathways. The highest enrichment score of pathways in upregulated mRNAs included the TNF signaling pathway, cytokine–cytokine receptor interaction pathway. For the downregulated mRNAs, the cell cycle pathway was included. This pathway is involved with myocardial infarction injury. The pathway enrichment for the genes in KEGG was analyzed using ClueGO (Fig. 5).

### LncRNA–mRNA co-expression network construction

**Fig. 6:**
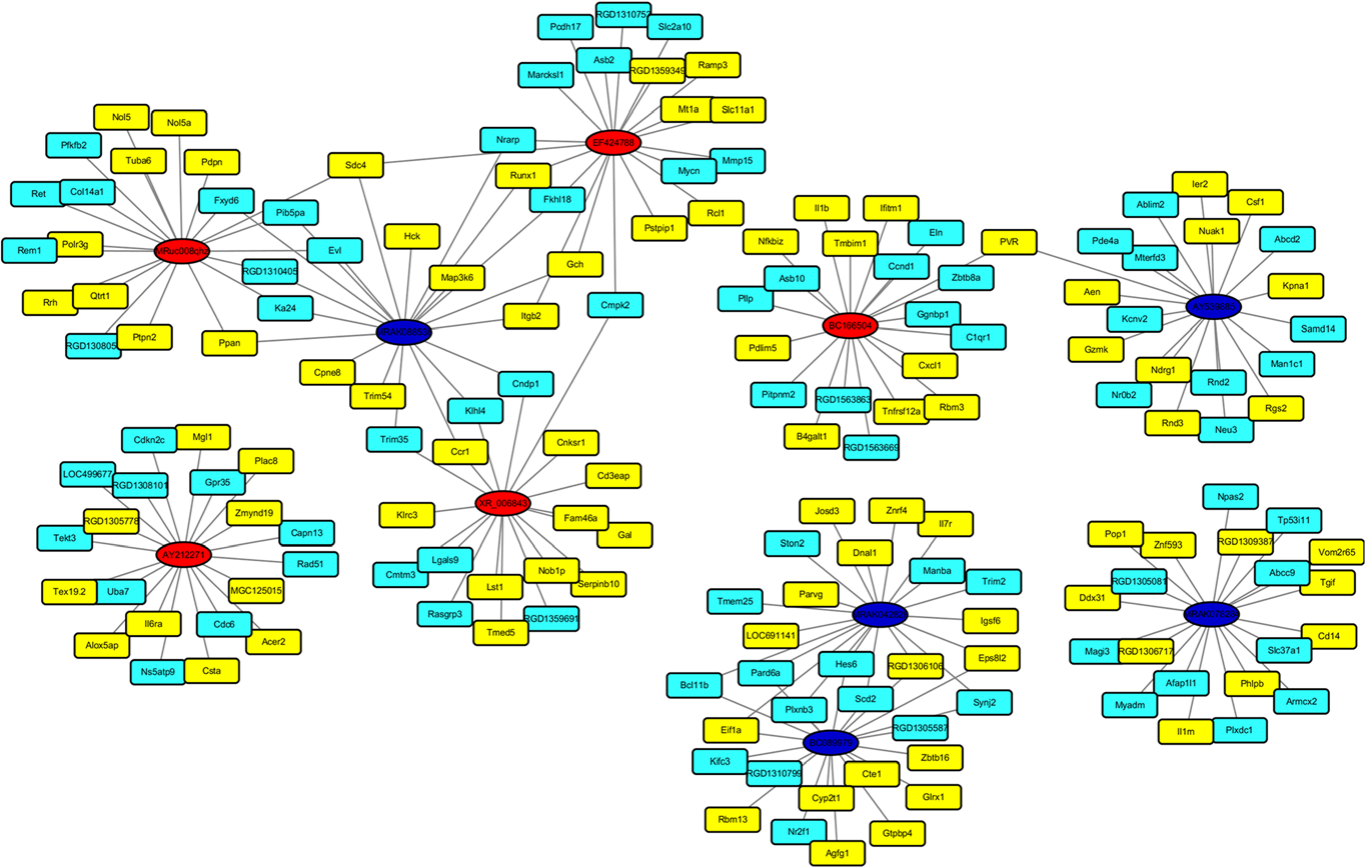
CNC-network. Red genes are upregulated lncRNAs. Deep blue genes are downregulated lncRNAs. Upregulated mRNAs are yellow and downregulated mRNAs are light blue

To investigate the relationship and the potential modulating mechanism between the aberrantly expressed mRNAs and the differentially expressed lncRNAs, we constructed a co-expression network. The 5 most significantly differentially expressed upregulated and downregulated lncRNAs were used to build the network. Based on the Pearson correlation coefficient (*R* > 0.99 or *R* < − 0.99, *p* < 0.01) between mRNAs and lncRNAs, we chose the top 10 upregulated and 10 downregulated co-expressed mRNAs for each lncRNA. The network containing the top 10 aberrantly expressed lncRNAs and the 198 most highly relevant dysregulated mRNAs is shown in Fig. 6.

### Confirmation of 10 highly dysregulated lncRNAs using quantitative RT-PCR

**Fig. 7:**
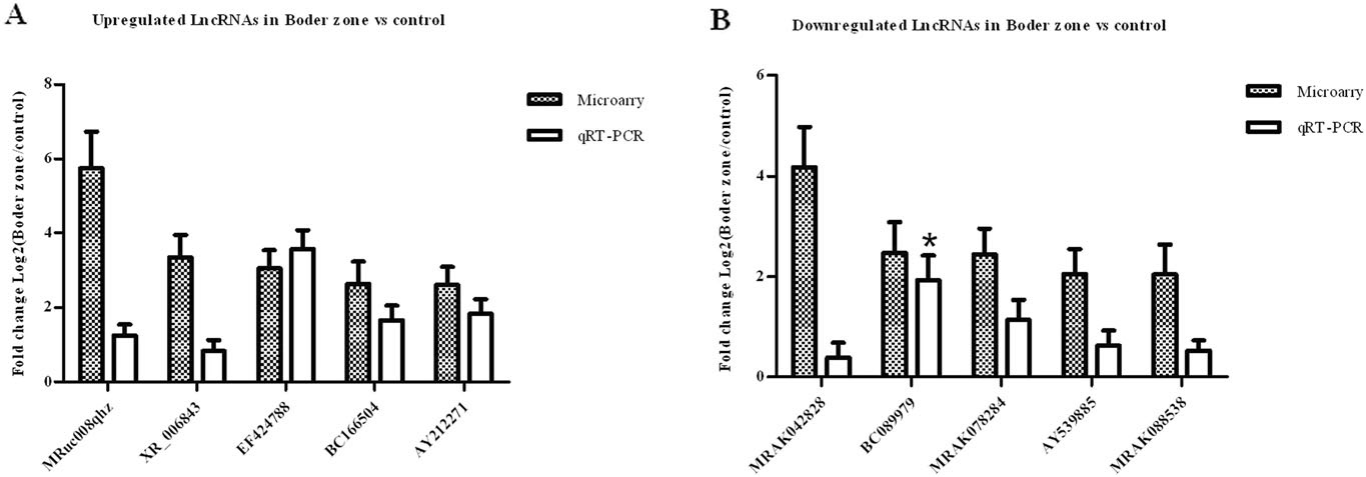
**a**: Upregulated lncRNAs in border zone detected by qRT-PCR vs microarry; **b**: Downregulated lncRNAs in border zone detected by qRT-PCR vs microarry. The height of the columns indicate the log-transformed fold changes in the expression between the border zone and the control zone, and the bars represent standard errors. The shaded columns present the microarry expression of lncRNAs, while the blank columns present the qRT-PCR results. *Indicates that there is a disagreement between the microarray data and quantitative RT-PCR result

To confirm the microarray results for the aberrantly expressed lncRNAs, quantitative RT-PCR was performed. We selected 10 lncRNAs for quantitative RT-PCR validation of their differential expression in the border zone of infarction. These lncRNAs were the most significantly dysregulated and all appeared in the gene co-expression network. The results of quantitative RT-PCR for the selected lncRNAs were generally consistent with the microarray data, except those for BC089979 (Fig. 7). The disagreement result could be acceptable because microarrays can sometimes generate false positive results.

## Discussion

Here, we present a global expression profiling of lncRNAs and mRNAs in the border zone of myocardial infarction. We also analyzed their potential biological functions.

The border zone decides the outcome of acute myocardial infarction, especially those reperfusion fails, with potential mechanisms of apoptosis, inflammation, LV remodeling and electric remodeling, and related processes [21–23]. Interestingly, the occlusion site of the coronary artery usually decides the size of the infarction area but not the size of the border zone (or the area at risk). For example, the areas were quite similar in patients with proximal and mid-left anterior descending coronary occlusions [1].

Therefore, the question is what decides the size of the border zone of myocardial infarction. To elucidate this, it is important to investigate whether and what role lncRNA plays in the border zone of myocardial infarction. We obtained border zone tissue very precisely through EB and TTC dual-dye staining. We found hundreds of lncRNAs and mRNAs that express differentially in border zone of myocardial infarction.

Unlike studies performed with mice models by microarray or RNA sequencing [10, 12], our results show that the total number of differentially expressed annotated lncRNAs in the border zone higher than the number in the myocardial infarction zone. This probably indicated that bioprocesses in the border zone were more active and more complicated. The number of upregulated lncRNAs in the border zone was greater than the downregulated number (407 vs 132), and this is different from the relative levels reported for the myocardial infarction zone. We considered this to indicate that in the border zone, more positive reactions were responding to the myocardial infarction than in the myocardial infarction zone itself.

In our study, some of the maximally dysregulated mRNAs, including Il6 and Ptx3 were directly related with myocardial infarction. Il6 is one of the inflammatory cytokines that participate in the inflammation response of myocardial infarction. Elevated Il6 levels are important risk markers and prognostic factors for myocardial infarction [24–26]; Il6 also contributes to the remodeling of the left ventricle after myocardial infarction [27]. Ptx3 shows a similar situation to Il6 [28–31]. Our results indicate that these changes to Il6 and Ptx3 reflect the inflammation response in the border zone, which is similar to the changes previously reported by other authors.

It should be noted that some aspects of our study restricted the results.. For example, we were short of biological repeats, and the microarray itself yielded some false positive results. Therefore, further studies were needed to confirm this information.

In the GO analysis, we found that the GO terms of the upregulated mRNAs in the border zone were most highly enriched in cytokine activity, binding, cytokine receptor binding and some related processes. Some of them, such as chemokine activity and chemokine receptor binding, were similar with the GO term changes in the myocardial infarction zone, but others were not [12].

In the pathway analysis, the upregulated mRNAs were mainly associated with inflammation, the immune and stress responses, cell proliferation, apoptosis and necrosis, and some related processes. The downregulated mRNAs were mainly associated with pathways involved in energy metabolism, cardiomyocyte hypertrophy, ion channels, apoptosis and growth, and some related processes. These results indicate that complicated compensation and decompensation occur in the border zone after myocardial infarction, and this showed the importance of protecting the border zone.

Recent research has found that lncRNAs may be important in regulating gene expression [32]. By constructing a co-expression network with aberrantly expressed protein-coding genes, we predicted the potential functions of lncRNAs. In the co-expression network, we found 16 genes are directly involved in myocardial infarction. For example, Alox5ap is reported to be involved in myocardial infarction with a degree of 49.27 (MalaCards score). This gene encodes a protein that is required for leukotriene synthesis together with 5-lipoxygenase and is implicate in various types of inflammatory response. Genetic variations in Alox5ap may be associated with susceptibility to myocardial infarction and stroke through an increase in leukotriene production and inflammation in the arterial wall [33–37]. The lncRNA AY212271 is co-expressed with Alox5ap. Therefore, we infer that AY212271 may participate in the inflammatory response in the border zone of myocardial infarction indirectly through Alox5ap.

Itgb2 co-expresses with both EF424788 and MRAK088538. Itgb2 is reported to be a risk factor of myocardial infarction and atherothrombotic cerebral infarction through inflammatory processes as a cell adhesion molecule [38–40]. Itgb2 is also involved in the reducing the risk of myocardial infarction due to adverse reactions to statins [41]. Therefore, we presume that one mRNA may be regulated by several lncRNAs at the same time, and that a single lncRNA can also affect several mRNAs simultaneously. For example, BC166504 co-expresses with 4 mRNAs involved in myocardial infarction: B4galt1, Eln, Il1b and Nfkbiz.

B4galt1 (beta-1,4-GalT-I) mRNA was mostly expressed in neutrophils, macrophages and endothelial cells. B4galt1 expression in the heart could be strongly induced by administration of LPS [42]. B4galt1 is also involved in the proliferation and apoptosis of Schwann cells induced by TNF-α via the activation of MAP kinase signal pathways [43]. The extracellular matrix (ECM) remodeling of the vessel wall is an important step in atherosclerosis and might potentially predict possible cardiovascular events. The elastin to collagen III ratio was significantly higher in aortic punch tissues from myocardial infarction patients [44]. Overexpression of Eln in the infarcted myocardium could attenuate scar expansion and improve heart function [45].

Il1b (interleukin-1 beta) is a key pro-inflammatory cytokine that has been associated with the development of atherosclerosis and myocardial infarction. Il1b gene polymorphisms influence the risk of myocardial infarction and ischemic stroke at a young age through NF-κB, iNOS, MMP-2 and Bax [46–48]. Controversially, there is lack of association between IL-1 gene polymorphisms and myocardial infarction in the Turkish population [49]. Il1b also activates a dexamethasone-sensitive myocardial L-arginine–NO pathway, which raises myocardial cyclic GMP and induces marked twitch aberration which leads to cardiac depression [50, 51].

Nfkbiz (nuclear factor-kappa B inhibitor zeta) is a nuclear inhibitor of NF-κB (IκB) protein. In myxoid liposarcoma, Nfkbiz plays a key role in inducing NF-κB-controlled genes deregulated by FUS-DDIT3 [52]. Nfkbiz controls the proliferation and differentiation of epidermal keratinocytes through NFκB-independent mechanisms [53]. Therefore, it is reasonable to presume that Nfkbiz may contribute to lowering myocardial infarction susceptibility through the potential reduction of activated NFкB, which is a key factor in inflammation [54].

All of the above indicates that BC166504 is probably a key lncRNA in the border zone of myocardial infarction, regulating inflammation, anti-inflammation, twitch aberration and scar attenuation via different pathways. More studies are needed to further confirm the functions of lncRNAs in the border zone of myocardial infarction.

## Conclusions

The study uncovered the underlying roles of lncRNAs in the border zone of myocardial infarction in rats. The results may evidence the underlying mechanisms of aberrantly expressed lncRNAs and mRNAs in the pathophysiology of myocardial infarction.

## Data Availability

We declare that materials described in the manuscript, including all relevant raw data, will be freely available to any scientist wishing to use them for non-commercial purposes, without breaching participant confidentiality.

## Abbreviations

B4galt1: Beta-1,4-GalT-I
BP: Biological process
CC: Cellular component
EB: Evans Blue dye
ECM: Extracellular matrix
FDR: False discovery rate
GO: Gene ontology
Il1b: Interleukin-1beta
IκB: Nuclear inhibitor of NF-κB
KEGG: Kyoto Encyclopedia of Genes and Genomes
LAD: Anterior descending artery
LncRNAs: Long noncoding RNAs
MF: Molecular function
mRNAs: Messenger RNAs
Nfkbiz: Nuclear factor-kappa B inhibitor zeta
PCC: Pearson’s correlation coefficient
TTC: Triphenyltetrazolium chloride
